# Physical activity promotes clinical benefits beyond dopaminergic preservation in Parkinson’s disease: a longitudinal analysis of the PPMI cohort

**DOI:** 10.1186/s42466-026-00518-z

**Published:** 2026-08-11

**Authors:** Minkyeong Kim, Kyoungjune Pak, Jae-Hyeok Lee, Myung Jun Lee

**Affiliations:** 1 Department of Neurology, Gyeongsang National University Hospital, Jinju, Republic of Korea; 2 Department of Nuclear Medicine, Pusan National University Hospital, Pusan National University School of Medicine and Biomedical Research Institute, Busan, Republic of Korea; 3 Department of Neurology, Pusan National University Yangsan Hospital, Pusan National University School of Medicine, Yangsan, Republic of Korea; 4 Department of Neurology, Pusan National University Hospital Pusan National University School of Medicine and Biomedical Research Institute, 179 Gudeok-ro, Seo-gu, Busan, 49241 Republic of Korea

**Keywords:** Parkinson’s disease, Physical activity, Dopamine transporter imaging, Motor symptoms, Cognition

## Abstract

**Background:**

Physical activity is associated with better clinical outcomes in Parkinson’s disease (PD). This study aimed to investigate the effects of physical activity on longitudinal changes in dopamine transporter (DAT) availability and motor and cognitive outcomes. Additionally, it explored whether striatal dopaminergic integrity mediates these effects.

**Methods:**

Using data from the Parkinson’s Progression Markers Initiative, we included patients with PD who had at least 4 years of follow-up data on the Physical Activity Scale for the Elderly and at least two assessments of DAT imaging, motor and cognitive functions, resulting in three analytical datasets (*n* = 141, 233, and 259, respectively). Linear mixed-effects models were used to analyse the influence of physical activity on the progression of DAT availability and clinical outcomes. Multilevel mediation analyses investigated the mediation effect of physical activity on the rate of clinical progression.

**Results:**

Higher levels of physical activity were significantly associated with a slower decline in caudate DAT availability, attenuated progression of axial symptoms, rigidity, and overall motor severity, and better preservation of Montreal Cognitive Assessment and Symbol Digit Modalities Test scores. Mediation analyses demonstrated that higher physical activity was significantly associated with slower caudate DAT decline; however, none of the clinical outcomes were significantly mediated by caudate DAT preservation, as all indirect effects were non-significant.

**Conclusion:**

These findings suggest that physical activity is associated with both dopaminergic degeneration and clinical progression in PD. However, the observed clinical benefits are likely primarily driven by mechanisms beyond striatal dopaminergic preservation.

**Supplementary Information:**

The online version contains supplementary material available at 10.1186/s42466-026-00518-z.

## Introduction

 Parkinson’s disease (PD) is characterised by progressive dopaminergic neuronal degeneration in the substantia nigra pars compacta [1]. These pathologic changes are associated with the hallmark motor features, including rigidity, bradykinesia, tremor, and gait disturbance [2]. Additionally, patients with PD often present with non-motor symptoms such as cognitive decline, depression, and orthostatic hypotension, which are attributed to the involvement of non-dopaminergic pathways [2, 3].

The current management of PD primarily focuses on symptomatic relief, using pharmacotherapy, deep-brain stimulation, and exercise. In addition to disease-modifying therapies targeting pathologic α-synuclein aggregation and drug repurposing, lifestyle factors have attracted growing interest as potential modifiers of disease manifestation and progression [4, 5]. In particular, enhanced physical activity—across diverse forms, intensities, and individual phenotypes—has been consistently associated with better clinical outcomes in PD [6, 7]. Higher levels of physical activity have been significantly associated with slower decline of postural and gait stability, activities of daily living, and processing speed [8]. Furthermore, initiating and maintaining exercise habits after diagnosis have been inversely associated with all-cause mortality in a dose-response manner [9]. Consequently, in clinical practice, interventions using wearable sensors and telemedicine have been implemented to promote physical activity within this population [10].

Physical activity has been suggested to help preserve dopaminergic neurons by enhancing neurotrophic factor expression, improving mitochondrial biogenesis, reducing neuroinflammation, and promoting autophagy, thereby suppressing pathological α-synuclein aggregation [11]. However, findings regarding the effects of physical activity on dopaminergic neurons have been inconsistent, varying according to disease stage, experimental models, and methods used to assess exercise exposure and dopaminergic function [12]. Furthermore, it remains unclear whether the preservation of dopaminergic neurons translates into clinical symptomatic benefit.

This study aimed to investigate the effect of regular physical activity on longitudinal changes in dopamine transporter (DAT) availability, motor function, and cognitive performance, extending previous findings by examining whether the clinical benefits of physical activity in PD are directly linked to the structural preservation of the dopaminergic system. We further examined whether the beneficial effects of regular physical activity on motor and cognitive functions may be mediated by the attenuated striatal dopaminergic function.

## Methods

### Study participants

This retrospective observational cohort study utilised data from the Parkinson’s Progression Markers Initiative (PPMI) database, downloaded on 18th December 2025. PPMI is an ongoing, multicenter, observational study that commenced in 2012. Briefly, PPMI has progressively enrolled patients with de novo PD who exhibit presynaptic dopaminergic terminal loss on DAT imaging and has conducted longitudinal follow-up assessments at 3- or 6-month intervals, with annual assessments thereafter. The detailed study protocol is available on the PPMI website (www.ppmi-info.org) [13].

To investigate the long-term effects of regular physical activity, patients with PD who had data from the Physical Activity Scale for the Elderly (PASE) questionnaire were included, with a minimum of 4 years between assessments. In addition, participants were required to have undergone at least two sets of DAT scans, as well as motor and cognitive evaluations, between their first and last PASE assessments. To maximize statistical power while maintaining analytical transparency, three distinct analytical datasets were constructed based on the availability of follow-up data (DAT imaging: *n* = 141; motor function: *n* = 233; cognitive function: *n* = 259, at baseline). Patients with PD and pathogenic variants in *glucocerebrosidase (GBA)*,* leucine-rich repeat kinase 2 (LRRK2)*,* parkin (PRKN)*,* alpha synuclein (SNCA)*, and *PTEN-induced kinase 1 (PINK1)* (*n* = 369), and those with negative findings on visual interpretation of DAT scans, were excluded (*n* = 31). The flowchart illustrating the construction of the three analytical datasets is provided in Supplementary Information 1.

In the original PPMI protocol, the PASE assessment was initially introduced at the 2-year follow-up visit [8, 14]. However, for participants who enrolled later in the study, PASE data were collected at earlier time points (e.g., at 1 year post-enrollment). Accordingly, the ‘baseline’ in this study was defined as the point at which the first PASE record was collected, regardless of the duration from enrolment.

### Physical activity measurement

Physical activity was assessed using the PASE, a self-report questionnaire that captures the intensity, frequency, and duration of physical activity over the preceding week [15]. The PASE comprises three domains: leisure, household, and work activities. The sum of these scores quantifies overall physical activity (https://meetinstrumentenzorg.nl/wp-content/uploads/instrumenten/PASE-handl.pdf). To estimate regular physical activity during the study period, the average total PASE score across the follow-up period was used [8].

### DAT image processing

DAT images acquired according to the PPMI imaging protocol were reconstructed using a standardised iterative reconstruction procedure and processed using MIAKAT software (version 5.0; XingImaging). Images were spatially normalised to MNI152 space, and regions of interest were applied using the CIC atlas. DAT availability was quantified as the specific binding ratio (SBR), calculated as (SUV_target_/SUV_reference_) -1, using the cerebral white matter and occipital cortex as reference regions [13]. In this study, the mean SBR values (averaged across both hemispheres) in the caudate nucleus and putamen were used.

### Clinical measurements

In addition to age at onset, sex, years of education, and disease duration at baseline, longitudinal data on motor and cognitive functions were collected. Regarding motor function, the “off” state Movement Disorder Society–sponsored revision of the Unified Parkinson’s Disease Rating Scale (MDS-UPDRS) Part III scores were utilised, defined as assessments performed after withholding dopaminergic medication for at least 12 h. Axial, tremor, rigidity, and bradykinesia subscores of MDS-UPDRS Part III were also calculated [16]. Hoehn-Yahr stage and Levodopa Equivalent Daily Doses (LEDDs) were additionally collected [17].

Global cognitive function was assessed using the Montreal Cognitive Assessment (MoCA) [18]. Specific cognitive domains were evaluated using the following battery: the Judgment of Line Orientation (JLO) for visuospatial function [19]; the Hopkins Verbal Learning Test-Revised (HVLT-R) immediate recall, delayed recall, and retention T-scores as a measure of verbal learning, episodic memory, and memory consolidation, respectively [20]; Semantic Fluency (animal naming) for semantic memory and executive function [21]; Letter Number Sequencing (LNS) for working memory and attention [22]; and the Symbol Digit Modalities Test (SDMT) for processing speed [23].

### Statistical analyses

The time points used for analysis and category of measurements are illustrated in Supplementary Information 2.

To explore the effects of regular physical activity on longitudinal changes in outcome variables, linear mixed-effects (LME) models with random slopes and intercepts were employed. To maximise statistical power and data utilisation, separate LME models were fitted for each outcome domain using the corresponding analytical datasets described above. In these models, the interaction term between time (years from baseline) and the average PASE score was included as the primary predictor of interest. Before analysis, continuous variables were standardised (z-scored). LME models were adjusted for age at onset, sex, disease duration at baseline, and LEDD. The models for cognitive dysfunction incorporated years of education.

For outcomes showing significant interactions with PASE in the primary LME models, further investigation was conducted to assess whether the rate of dopaminergic degeneration (DAT SBRs slopes) mediated the association between regular physical activity (average PASE score) and the rate of clinical progression (motor or cognitive slopes). Individualised DAT slopes (mediator) were extracted as Best Linear Unbiased Predictions from LME models using data from participants with complete records (*n* = 127 and 135, for motor and cognitive progression, respectively). Subsequently, multilevel mediation analyses using LME models were conducted, where clinical progression was operationalised as the interaction term between time and the predictor variables. Clinical variables were standardised, and the models were adjusted for the same covariates stated above. The significance of effects in the mediation analyses was assessed using the Monte Carlo method with 20,000 simulations to construct 95% confidence intervals (CIs) [24].

To examine the robustness of our findings, several sensitivity analyses were conducted. First, to address the possibility of reverse causation, a control analysis was performed, in which baseline DAT SBRs were used as predictors of longitudinal changes in PASE scores using LME models, adjusted for the same covariates as the primary analyses. Second, longitudinal changes in PASE scores over the follow-up period were investigated and an additional LME model was performed using PASE scores at each assessment timepoint as predictor. Third, to explore potential selective attrition, baseline characteristics were compared between participants included in our analysis (those with ≥ 48 months of PASE follow-up, *n* = 257) and all remaining PD participants registered in the PPMI dataset (*n* = 1,262), using Mann-Whitney U tests with rank-biserial correlation as the effect size measure.

Histograms, Q-Q plots, and partial regression plots demonstrated that although minor deviations from normality were observed in the residuals, these were considered acceptable given the large sample sizes, which mitigate the impact of non-normality on parameter estimates (Supplementary Information 3).

Continuous variables are presented as mean ± standard deviation, and categorical variables are expressed as frequencies. To account for multiple comparisons, *p*-values were adjusted using the Benjamini-Hochberg false discovery rate (FDR) procedure. Unless otherwise specified, all reported *p*-values denote FDR-corrected values. Statistical significance was defined as *p*-value < 0.05. All statistical analyses were conducted using Python (version 3.12.12) and R software (version 4.4.3; R Foundation for Statistical Computing, Vienna, Austria), with the *lme4*, *lmerTest*, and *emmeans* packages [25–27].

## Results

### Clinical characteristics of the study participants

Demographic and clinical characteristics at baseline are summarised in Table 1, and temporal changes in clinical parameters are presented in Supplementary Information 4.

**Table 1:** Baseline characteristics of the enrolled participants

	Analytical datasets		
	DAT imaging (*n* = 141)	Motor function (*n* = 233)	Cognition function (*n* = 259)
Age at onset, y	58.28 ± 9.64	59.06 ± 9.76	59.30 ± 9.70
Sex (M: F)	95 : 46	164 : 69	180 : 79
Disease duration at baseline, y	3.44 ± 1.76	3.76 ± 2.08	3.79 ± 2.06
PPMI enrollment to baseline, y	1.55 ± 0.57	1.84 ± 0.79	1.87 ± 0.79
PASE scores	170.74 ± 83.75	172.84 ± 93.46	176.21 ± 98.64
LEDD	287.32 ± 282.15	317.37 ± 304.80	330.04 ± 295.29
Missing, n^*^	10	11	19
Education, y			15.89 ± 2.70
Caudate DAT SBRs	0.80 ± 0.26		
Putamen DAT SBRs	0.65 ± 0.22		
“off” MDS-UPDRS Part III			
Axial		2.65 ± 2.11	
Rigidity		5.16 ± 3.05	
Bradykinesia		12.72 ± 6.29	
Tremor		5.52 ± 4.00	
Total		26.05 ± 10.77	
HY stage		1.78 ± 0.52	
MoCA			26.51 ± 2.65
JLO			12.74 ± 2.29^†^
HVLT-R recall			46.41 ± 11.16
HVLT-R delayed recall			45.96 ± 11.41
HVLT-R retention			47.78 ± 11.74
Semantic Fluency (animal)			21.48 ± 5.45
LNS			10.58 ± 2.66
SDMT			41.45 ± 10.05

DAT = dopamine transporter; PPMI = Parkinson’s Progression Markers Initiative; PASE = Physical Activity Scale for the Elderly; LEDD = levodopa equivalent daily dose; SBRs = specific binding ratios; MDS-UPDRS Part III = Movement Disorder Society sponsored Unified Parkinson’s Disease Rating Scale part III; HY stage = Hoehn and Yahr stage; MoCA = Montreal Cognitive Assessment; JLO = Judgment of Line Orientation; HVLT-R= Hopkins Verbal Learning Test-Revised; LNS = Letter Number Sequencing; SDMT = Symbol Digit Modalities Test. Data are presented as mean ± standard deviation or numbers

^*^LEDD data were missing at baseline for 10, 11, and 19 participants in the DAT imaging, motor, and cognitive function datasets, respectively. Participants with missing LEDD at baseline but available LEDD at subsequent timepoints were retained in longitudinal analyses

^†^One participant had missing data for JLO assessments at baseline

The results of control analyses, time effects in LME models, LME models using time-varying PASE as a predictor, and selective attrition analyses are presented in Supplementary Information 5 through 8, respectively.

### Effects of baseline DAT availability on the progression of clinical parameters

First, to address the potential for reverse causality, a control analysis revealed that neither caudate nor putaminal DAT SBRs at baseline predicted the rate of change in PASE scores over time (Supplementary Information 5). In the LME models, time effects were significant for all DAT and motor variables (all FDR-corrected *p* < 0.05), indicating progressive decline over the follow-up period. Most cognitive variables also showed significant declining trends; however, MoCA, HVLT-R recall, and HVLT-R delayed recall did not exhibit a significant mean change at the group level (Supplementary Information 6). Regarding interaction effects, higher average PASE scores were associated with a significantly attenuated annual decline in caudate DAT SBRs (estimate = 3.769 × 10^− 2^, *p* = 0.041), whereas no significant association was observed in the putamen. Higher average PASE scores were associated with a significantly slower worsening of MDS-UPDRS Part III axial subscores (estimate = − 5.679 × 10^− 2^, *p* < 0.001), rigidity subscores (estimate = − 2.741 × 10^− 2^, *p* = 0.042), and total scores (estimate = − 3.113 × 10^− 2^, *p* = 0.026). Bradykinesia and tremor subscores demonstrated no significant associations (Table 2).

**Table 2:** Effects of physical activity on the progression of DAT availability, motor, and cognitive outcomes

	Estimate(×10^-^^2^)	SE(×10^-2^)	p	Corrected-p^*^
DAT SBRs				
Caudate	3.769	1.611	0.020	0.041^†^
Putamen	0.612	1.791	0.733	0.733
"off" MDS-UPDRS Part III				
Axial	-5.679	1.235	< 0.001	< 0.001^†^
Rigidity	-2.741	1.214	0.025	0.042^†^
Bradykinesia	-1.550	1.151	0.179	0.224
Tremor	-0.310	1.296	0.811	0.811
Total	-3.113	1.207	0.011	0.026^†^
Cognitive tests				
MoCA	2.838	1.038	0.007	0.027^†^
JLO	0.953	1.030	0.356	0.406
HVLT-R recall	0.713	1.011	0.481	0.481
HVLT-R delayed recall	0.991	1.015	0.330	0.406
HVLT-R retention	1.424	1.162	0.222	0.354
Semantic Fluency (animal)	1.379	0.988	0.164	0.328
LNS	2.019	0.960	0.037	0.097
SDMT	2.739	0.840	0.001	0.010^†^

DAT = dopamine transporter; SE = standard error; SBRs = specific binding ratios; MDS-UPDRS Part III = Movement Disorder Society–sponsored Unified Parkinson's Disease Rating Scale part III; MoCA = Montreal Cognitive Assessment; JLO = Judgment of Line Orientation; HVLT-R = Hopkins Verbal Learning Test-Revised; LNS = Letter Number Sequencing; SDMT = Symbol Digit Modalities Test.^*^ False discovery rate (FDR) correction using the Benjamini–Hochberg procedure was applied separately within each domain.^†^ Statistical significance was defined as FDR-adjusted p < 0.05.

**Table 3:** Mediation analysis: Role of Caudate DAT SBR in the association between physical activity and clinical progression

	Path A^a^ (PASE → DAT)	Path B(DAT → Outcome)	Indirect(PASE → DAT → Outcome)	Direct(PASE → Outcome)	Total Effect
Motor^b^(n = 127)					
Axial	0.204 (0.022, 0.385)	−0.020 (−0.050, 0.011)	−0.004 (−0.013, 0.002)	−0.031 (−0.063, 0.001)	−0.036 (−0.067, −0.005)
	p = 0.030^*^	p = 0.205	p = 0.232	p = 0.058	p = 0.024^*^
Rigidity	0.204 (0.022, 0.385)	−0.017 (−0.048, 0.015)	−0.003 (−0.013, 0.003)	−0.023 (−0.056, 0.009)	−0.027 (−0.059, 0.004)
	p = 0.030^*^	p = 0.301	p = 0.325	p = 0.163	p = 0.090
Total	0.204 (0.022, 0.385)	−0.019 (−0.051, 0.013)	−0.004 (−0.013, 0.003)	−0.013 (−0.047, 0.020)	−0.018 (−0.051, 0.014)
	p = 0.030^*^	p = 0.242	p = 0.269	p = 0.431	p = 0.270
Cognition (n = 135)					
MoCA	0.215 (0.038, 0.391)	0.006 (−0.015, 0.027)	0.001 (−0.004, 0.007)	0.008 (−0.015, 0.031)	0.010 (−0.012, 0.032)
	p = 0.019^*^	p = 0.593	p = 0.604	p = 0.477	p = 0.382
SDMT	0.215 (0.038, 0.392)	0.016 (−0.003, 0.036)	0.004 (−0.001, 0.010)	0.021 (0.000, 0.043)	0.026 (0.005, 0.047)
	p = 0.019^*^	p = 0.108	p = 0.124	p = 0.054	p = 0.016^*^

PASE = Physical Activity Scale for the Elderly; DAT = dopamine transporter; SBR = specific binding ratio; MoCA = Montreal Cognitive Assessment; SDMT = Symbol Digit Modalities Test.^a^Path A represents the effect of PASE on the rate of change in Caudate DAT SBR. Since the exposure (PASE) and mediator (Caudate DAT SBR) are identical across outcomes within the same domain (Motor or Cognition), Path A estimates are largely constant across outcomes within each domain. Minor numerical differences reflect variations in the number of valid observations due to outcome-specific missing data.^b^Movement Disorder Society–sponsored Unified Parkinson's Disease Rating Scale Part III subscores. Data are presented as standardised regression coefficients with 95% confidence intervals (β [95% CI]) and p-values. ^*^ p < 0.05.

In the cognitive domain, higher average PASE scores predicted a significantly slower rate of decline in MoCA (estimate = 2.838 × 10^− 2^, *p* = 0.027) and SDMT (estimate = 2.739 × 10^− 2^, *p* = 0.010). Although LNS demonstrated nominal significance, this association was not robust after FDR correction. JLO, HVLT-R immediate recall, delayed recall, and retention, and Semantic Fluency showed no significant association (Table 2).

**Fig. 1:**
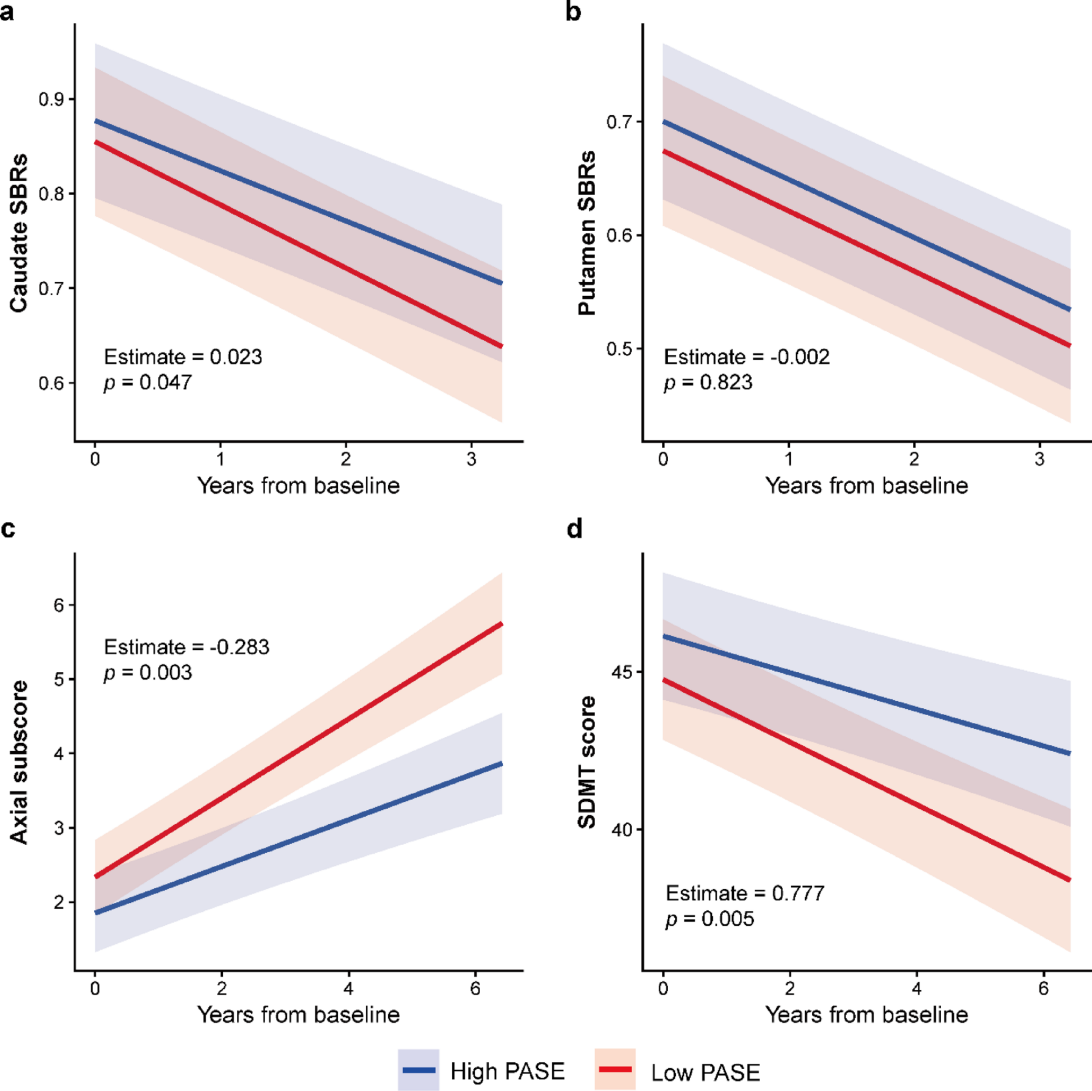
Longitudinal trajectories of DAT SBR, motor, and cognitive scores according to physical activity levels. The panels illustrate the predicted trajectories of (**a**) caudate DAT SBR, (**b**) putamen DAT SBR, (**c**) “off” MDS-UPDRS Part III axial subscores, and (**d**) SDMT scores according to physical activity levels. Lines represent the linear predicted trends for the High (blue, ≥ 75percentile) and Low (red, ≤ 25 percentile) PASE groups, derived from LME models. Shaded areas indicate 95% confidence intervals. The estimates represent the difference in the annual rate of change (slope) between the two groups (Time × Group interaction term)

For visualisation purposes, patients with PD were categorised into High (≥ 75percentile) and Low (≤ 25 percentile) PASE groups based on average PASE scores over the follow-up period. Comparison of the longitudinal trajectories between the High and Low PASE groups revealed that the High physical activity group exhibited a significantly slower rate of decline in caudate DAT SBRs and SDMT scores, as well as slower progression of MDS-UPDRS Part III axial subscores, compared with the Low physical activity group (Fig. 1).

## Discussion

This study, utilising the PPMI cohort, investigated the effects of regular physical activity on longitudinal changes in dopaminergic availability and motor and cognitive outcomes in PD patients. As our study has an observational design, causal inference between physical activity and clinical outcomes cannot be established. However, baseline DAT SBRs were not associated with longitudinal changes in PASE scores over time, suggesting that the severity of DAT deficits at baseline may not attenuate physical activity habits over time, supporting the directionality of our analyses.

Numerous clinical trials have demonstrated that exercise interventions have beneficial effects on both motor and non-motor symptoms and on quality of life in PD [28]. The findings from this study extend these observations by showing that overall physical activity—encompassing non-exercise daily activities—is associated with better preservation of caudate DAT availability and a slower progression of axial symptoms, rigidity, and overall motor severity, as well as MoCA and SDMT scores. However, mediation analyses revealed that the clinical benefits of physical activity were not significantly mediated by dopaminergic preservation, suggesting that these benefits may be driven by alternative, non-dopaminergic systems, such as the serotonergic, noradrenergic, or GABAergic systems, or those involving neurotrophic factors.

Beyond symptomatic improvement, whether physical activity or exercise reverses dopaminergic neuronal loss has been extensively investigated. In animal models of PD, treadmill exercise has been shown to inhibit dopaminergic neuronal loss in the substantia nigra, reduce pathologic p-α-synuclein accumulation, and increase DAT expression [29, 30]. A recent pilot study of early-stage PD (with an average disease duration of 2 years) reported that 6 months of high-intensity exercise attenuated the expected decline in DAT availability in the striatum and substantia nigra, demonstrating a significantly better outcome than the natural disease course [31].

In healthy older adults, physical activity has been associated with dopamine receptor availability assessed by [^11^C]raclopride positron emission tomography, suggesting that physical activity may influence dopaminergic signaling even in the absence of neurodegeneration [32, 33]. In this study, higher physical activity was associated with slower decline in caudate DAT availability, but not in the putamen. Given that dopaminergic degeneration is more pronounced and progresses more rapidly in the dorsal putamen—where comprehensive loss of dopamine markers is observed within 4 years of disease duration—than in the caudate nucleus [34], and considering that participants in our cohort had a mean disease duration of 3.44 years at baseline, the detection of potential protective effects of physical activity in putamen may have been limited. Furthermore, as the study assessed overall physical activity rather than a high-intensity exercise intervention, it is possible that earlier initiation or higher exercise intensity may be required to induce detectable structural changes in dopaminergic neurons.

A recent study revealed that PASE scores were significantly associated with motor reserve —defined as difference between observed and predicted motor symptoms based on putamen DAT SBRs— and that higher PASE scores were associated with a slower rate of decline in motor reserve [35]. This finding suggests that the beneficial effects of physical activity on motor symptoms may operate through extra-dopaminergic systems, such as compensatory cortico-striatal circuits or enhanced neuromuscular coupling, rather than through measurable changes of dopaminergic integrity. Notably, a previous study has reported that patients with PD and prominent axial motor symptoms showed less improvement in motor severity after 24 weeks of exercise intervention, suggesting that the responsiveness to exercise may vary by disease stage and clinical subtype [7].

The caudate nucleus plays a central role in cognitive and emotional processes within the frontostriatal circuit [36], and is linked to both motor and non-motor features of PD. A functional magnetic resonance imaging study involving 56 patients with PD revealed a correlation between the severity of gait disturbance and functional connectivity of the caudate nucleus [37]. In addition, PPMI data demonstrated that early caudate DAT loss predicted subsequent cognitive impairment and gait disturbance in PD [38]. Collectively, the preservation of caudate dopaminergic integrity may contribute to the maintenance of both motor and cognitive functions. However, in the present study, mediation analyses showed that caudate DAT preservation did not mediate the effect of regular physical activity on either motor and cognitive outcomes. These findings suggest that the clinical benefits of regular physical activity are not fully elucidated by striatal dopaminergic preservation and likely involve alternative mechanisms beyond the dopaminergic system.

Recent studies have proposed that the beneficial effects of physical activity may be explained by the concept of motor reserve [39, 40]. Patients with PD who engage in more physical activity show better motor performance despite similar dopaminergic degeneration, suggesting enhanced resilience within non-dopaminergic networks [41]. PPMI data demonstrated that appropriate medication and regular physical activity were associated with the maintenance of motor reserve [35]. Axial symptoms, which were significantly attenuated by physical activity in this study, are linked to the cholinergic system, including striatal cholinergic interneurons, the pedunculopontine nucleus, and the entorhinal cortex, and are largely independent of dopaminergic deficit [42, 43]. Similarly, while rigidity is primarily linked to dopaminergic denervation, alterations in transcortical or spinal long-latency reflex pathways contribute to its pathophysiology and are regulated by the noradrenergic and serotonergic systems [44]. Given that the extra-basal ganglia network, including frontal, limbic, and thalamic regions, has been associated with motor reserve in drug-naïve early PD [40], physical activity may promote motor reserve by enhancing these non-dopaminergic networks.

This study has some limitations. First, physical activity was assessed using the PASE questionnaire, which reflects activity over the preceding week; thus, it may not fully capture habitual physical activity patterns over the long term. Nevertheless, it is a well-validated tool that has shown high correlation with objective measures of physical activity [45].

Additionally, using average PASE may mask changes in physical activity levels over time. Although PASE scores showed a gradual decline over the follow-up period (Supplementary Figure S7-1), the large inter-individual variability in trajectories supports the use of average scores to characterise each participant’s habitual physical activity level. The use of average PASE scores across the follow-up period was further validated by a sensitivity analysis using time-varying PASE scores at each assessment timepoint, which yielded largely consistent findings (Supplementary Table S7-1), strengthening the validity of our findings.

Second, as DAT imaging was primarily obtained at the baseline and at years 1, 2, and 4 in the original PPMI protocol, it was not possible to assess effects beyond this period. Although DAT imaging is a useful diagnostic tool, its utility as a long-term biomarker may be limited, as DAT binding declines exponentially, and the motor manifestations of PD typically emerge after at least 50% loss of DAT binding [46]. Future studies integrating biofluid biomarkers, such as amyloid β, tau, and α-synuclein, are warranted to provide a more comprehensive understanding of the mechanisms underlying the effects of physical activity in PD.

Third, an analysis of potential selection bias revealed that included participants were slightly younger at symptom onset, had better baseline DAT availability, motor and cognitive functions on several measures compared to excluded participants (Supplementary Information 8). However, all statistically significant differences demonstrated small effect sizes (|r| ≤ 0.20), implying limited practical significance. These results suggest that while some degree of bias is present, it is unlikely to have substantially affected the primary findings. Nevertheless, the inclusion of participants with relatively milder disease may limit the generalisability of our findings, warranting further research in patients with more advanced PD.

In conclusion, this study suggests that although regular physical activity attenuates the caudate dopaminergic degeneration and motor and cognitive progression in PD, the observed clinical benefits appear to be primarily driven by mechanisms independent of striatal dopaminergic preservation. These findings highlight the importance of regular physical activity as a therapeutic strategy to enhance functional compensation, even in the context of progressive dopaminergic degeneration.

### Mediation analysis

**Fig. 2:**
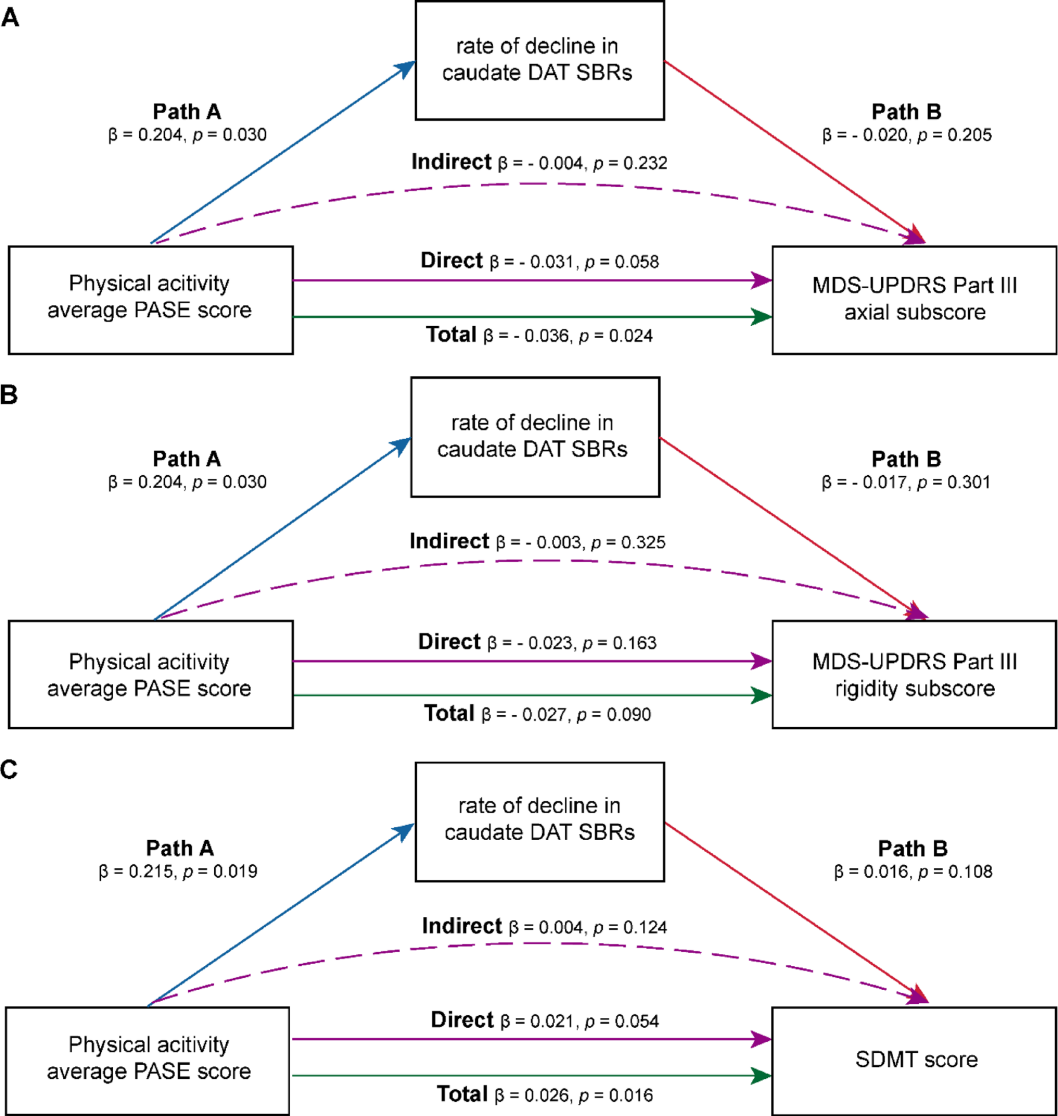
Mediation analysis results for motor and cognitive outcomes. Path diagrams illustrate the mediating role of DAT imaging (caudate slope) in the relationship between physical activity (average PASE score) and clinical outcomes. The dependent variables were the rate of change in (**a**) MDS-UPDRS Part III axial subscores, (**b**) rigidity subscores, and (**c**) SDMT scores. In all models, the average PASE score was the independent variable, and the rate of decline in caudate DAT SBRs was the mediator. Path A (blue line) represents the effect of physical activity on caudate DAT SBR changes, and Path B (red line) represents the effect of caudate DAT SBR changes on the clinical outcome. The total effect (green line) represents the overall association, which consists of the indirect effect (dashed purple line) and the direct effect (solid purple line). Standardised regression coefficients (β) and p-values are indicated for each path. DAT, dopamine transporter; SBR, specific binding ratio; PASE, Physical Activity Scale for the Elderly; MDS-UPDRS III, Movement Disorder Society-sponsored Unified Parkinson’s Disease Rating Scale Part III; SDMT, Symbol Digit Modalities Test.

As shown in Fig. 2; Table 3, Path A analyses demonstrated that higher average PASE scores were directly associated with a slower rate of decline in caudate DAT availability across both motor (β = 0.204, 95% CI [0.022, 0.385], *p* = 0.030) and cognitive outcome analyses (β = 0.215, 95% CI [0.038, 0.391], *p* = 0.019). However, the indirect effects of PASE via caudate DAT availability were not statistically significant for all clinical outcomes. Regarding total effects, PASE was significantly associated with slower progression of axial motor subscores (β = −0.036, 95% CI [− 0.067, − 0.005], *p* = 0.024) and better maintenance of SDMT (β = 0.026, 95% CI [0.005, 0.047], *p* = 0.016), consistent with the primary LME findings.

## Supplementary Information

Below is the link to the electronic supplementary material.

Supplementary Material 1

Supplementary Material 2

Supplementary Material 3

Supplementary Material 4

Supplementary Material 5

Supplementary Material 6

Supplementary Material 7

Supplementary Material 8

## Data Availability

All data analysed in the present study are available in the PPMI database. The R and Python codes used in this study are available upon authorised request.
