# Supplementary material for: Physical activity promotes clinical benefits beyond dopaminergic preservation in Parkinson’s disease: a longitudinal analysis of the PPMI cohort
Source: Neurol Res Pract. 2026 Aug 11;8(1):64. doi: 10.1186/s42466-026-00518-z (PMC13463522; doi:10.1186/s42466-026-00518-z)

Supplementary Information 3. Histograms, QQ plots, and partial regression plots

Figure S3−1. DAT imaging dataset

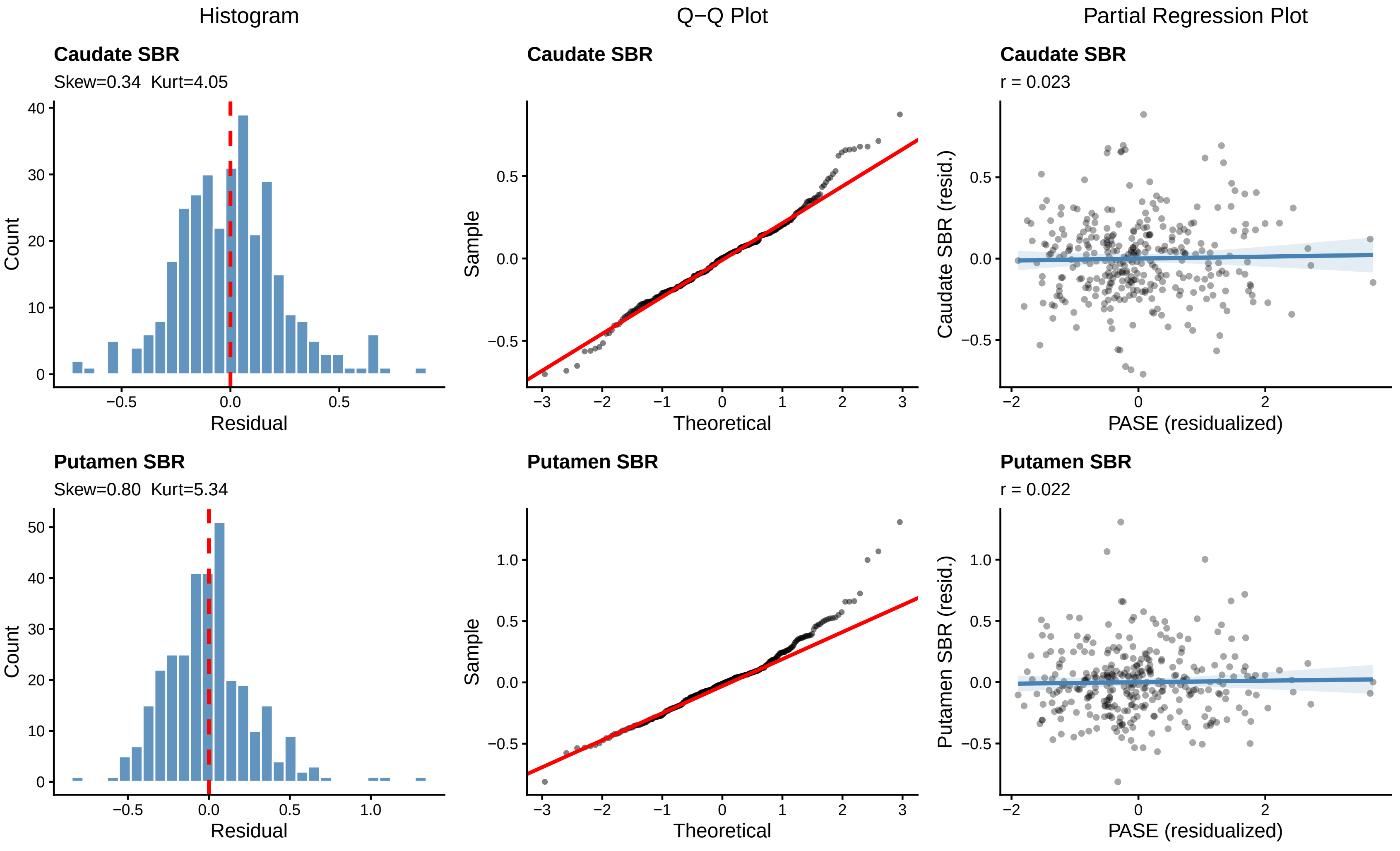

Figure S3−2. Motor function dataset

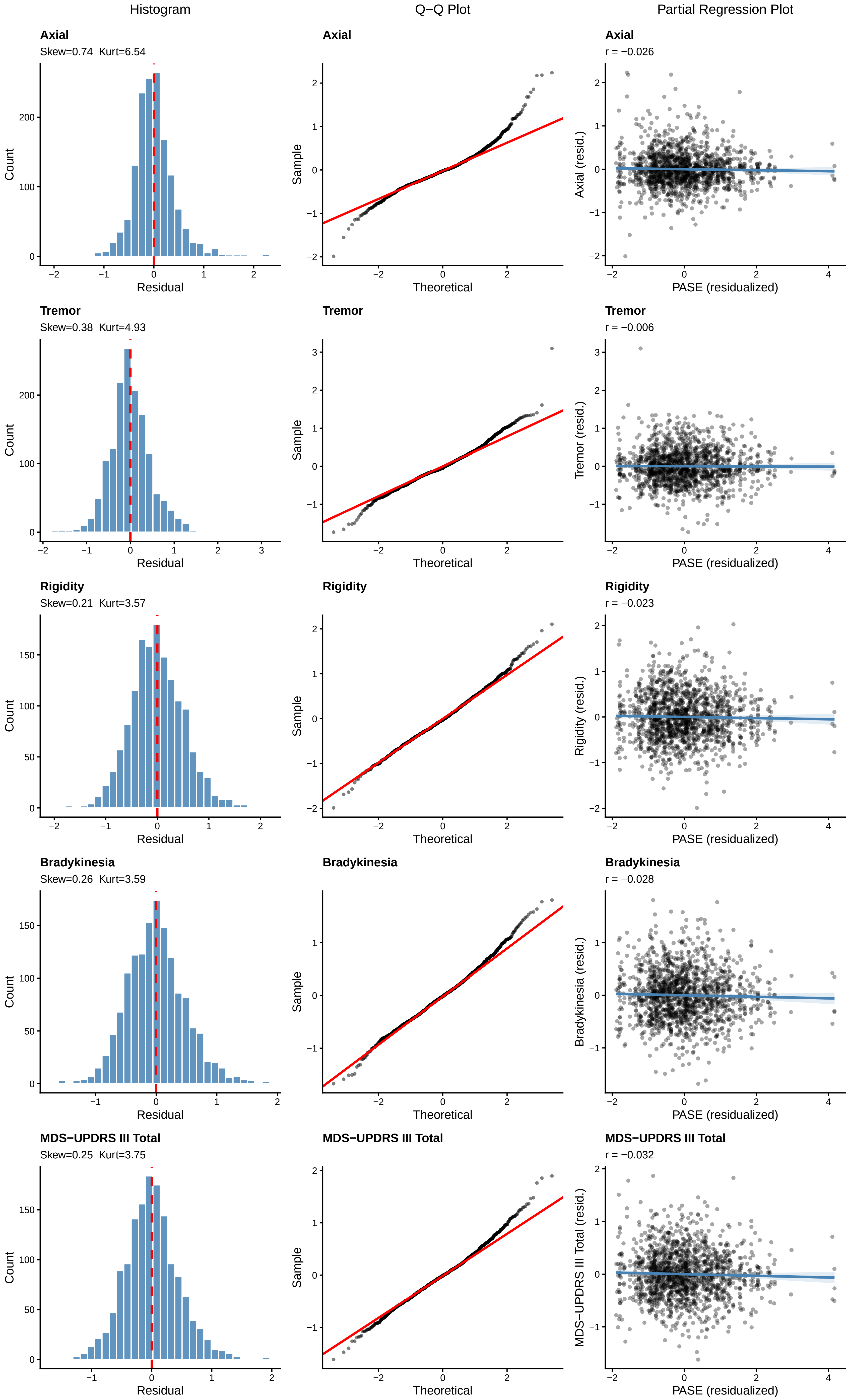

Figure S3−3. Cognitive function dataset

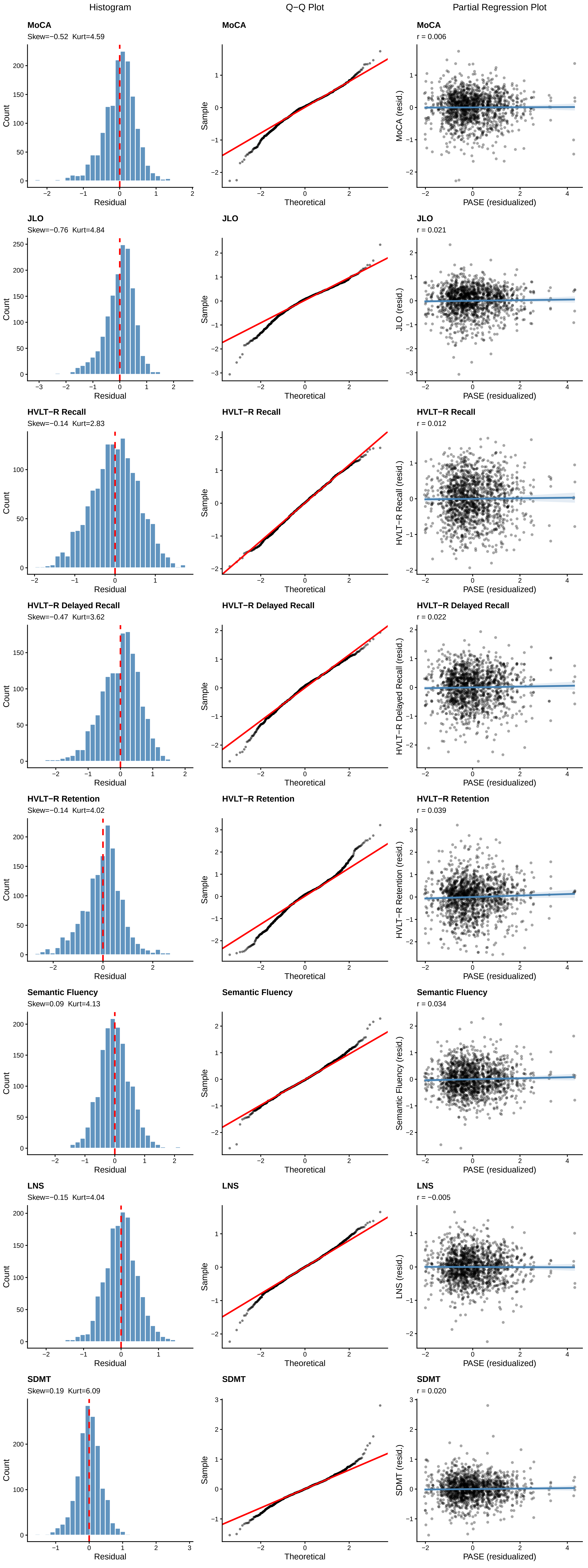

Supplement: Supplementary file 1 [file 42466_2026_518_MOESM3_ESM.pdf]
